# Three New Species of *Penicillium* from East and Northeast China

**DOI:** 10.3390/jof10050342

**Published:** 2024-05-08

**Authors:** He Song, Yi-Jing Ding, Wen-Ying Zhuang, Guang-Zhou Ding, Xin-Cun Wang

**Affiliations:** 1 College of Modern Agriculture and Ecological Environment, Heilongjiang University, Harbin 150080, China; 13104631380@163.com; 2 College of Life Science, Capital Normal University, Beijing 100048, China; 1200802007@cnu.edu.cn; 3 State Key Laboratory of Mycology, Institute of Microbiology, Chinese Academy of Sciences, Beijing 100101, China; zhuangwy@im.ac.cn

**Keywords:** Aspergillaceae, biodiversity, Eurotiales, phylogeny, taxonomy

## Abstract

*Penicillium* species are ubiquitous in the environment and are of substantial importance, especially in industrial and medical aspects. During our investigation of the biodiversity of *Penicillium*, three new species were discovered in soil samples collected from East and Northeast China. They were determined as new to science based on morphological comparisons and phylogenetic analyses, and were found to belong to the subgenus *Penicillium* section *Robsamsonia* and subgenus *Aspergilloides* sections *Aspergilloides* and *Citrina*. Descriptions and illustrations of these species are provided, and their geographic distributions are also discussed.

## 1. Introduction

Species of *Penicillium* Link are ubiquitous. Some of them are of industrial and medical importance. A range of efficient plant-polysaccharide-degrading enzymes are secreted by *Penicillium* species, such as *P. oxalicum* Currie & Thom, and are very useful for sustainable bioproduction [1]. *Penicillium sumatraense* Svilv. has the potential to be adopted in algal bio-refinery processes for biofuel production because of its arsenal of degrading enzymes [2]. Enzymes from *Penicillium* species are also crucial for the enhanced saccharification of agro-industrial wastes, which can be used to build a bio-based economy [3]. In the medicinal field, the best-known antibiotic penicillin was produced by *P. chrysogenum* Thom [4]. And more than 280 compounds have been reported from this genus, exhibiting lots of bioactive effects, e.g., antimicrobial, anticancer, antiviral, and antioxidant effects [5]. On the other hand, *P. digitatum* (Pers.) Sacc. is not only a major source of postharvest decay in citrus fruits worldwide [6] but is also a rare human pathogen that can cause fatal pneumonia in immunocompromised hosts [7,8].

The genus *Penicillium* was established in 1809, and *P. expansum* Link was designated as the type species. It is the most speciose in the order Eurotiales. In a monography published in 2014, 354 species were accepted in this genus [9], and 483 species were recognized in one that was published in 2020 [10]. By the end of 2022, 64 species had been further added to this group [11]. In the last year, 54 new species were described, and 43 of them were discovered in Southwestern China [12]. This leads to the species number of the genus over 600 at this moment. In China, more than 170 *Penicillium* species have been recorded, of which 91 were originally described from this country [12].

During an investigation into the biodiversity of *Penicillium*, three new species were discovered from the soil samples collected in East and Northeast China. Their descriptions and illustrations are provided here.

## 2. Materials and Methods

### 2.1. Fungal Materials

Cultures were isolated from soil samples collected from Heilongjiang, Jiangsu and Shanghai provinces or province-level municipality during 2021 to 2023. Dried cultures were deposited in the Herbarium Mycologicum Academiae Sinicae (HMAS, Beijing, China), and the living ex-type strains were preserved at the China General Microbiological Culture Collection Center (CGMCC, Beijing, China).

### 2.2. Morphological Observations

Morphological characterization was conducted following standardized methods [9]. Four standard growth media were used: Czapek yeast autolysate agar (CYA, yeast extract Oxoid, Hampshire, UK), malt extract agar (MEA, Amresco, Solon, OH, USA), yeast extract agar (YES), and potato dextrose agar (PDA). The methods of inoculation, incubation, microscopic examination, and digital recording matched those described in our previous studies [12,13,14,15,16,17,18].

### 2.3. DNA Extraction, PCR Amplification, and Sequencing

DNA was extracted from the cultures grown on PDA for 7 days using the Plant Genomic DNA Kit (DP305, TIANGEN Biotech, Beijing, China). Polymerase chain reaction (PCR) amplifications of the internal transcribed spacer (ITS), beta-tubulin (BenA), calmodulin (CaM), and RNA polymerase II second largest subunit (RPB2) gene regions were conducted using the routine methods [9]. The products were purified and subjected to sequencing on an ABI 3730 DNA Sequencer (Applied Biosystems, Foster, CA, USA). Although the ITS region, the proposed universal DNA barcode for fungi, is helpful to classify a *Penicillium* species at the section or series level, it is not sufficient to distinguish them at species level. However, ITS sequences are still provided here, as they might be beneficial to other researchers.

### 2.4. Phylogenetic Analyses

The forward and reverse sequences newly generated in this study were assembled using Seqman v. 7.1.0 (DNASTAR Inc., Madison, WI, USA). The assembled sequences were deposited in GenBank. The sequences used for phylogenetic analyses are listed in Table 1, Table 2 and Table 3. Sequences of each of the three single gene datasets (BenA, CaM and RPB2) and the concatenated ones were aligned using MAFFT v. 7.221 [19], then manually edited and concatenated in BioEdit v. 7.1.10 [20] and MEGA v. 11.0.13 [21]. Maximum likelihood (ML) analyses were conducted using RAxML-HPC2 [22] on XSEDE 8.2.12 on CIPRES Science Gateway v. 3.3 [23] with the default GTRCAT model and bootstrap (BP) iteration setting. Bayesian inference (BI) analyses were performed with MrBayes v. 3.2.7 [24]. Appropriate nucleotide substitution models and parameters were determined using Modeltest v. 3.7 [25]. Four MCMC chains were run for at least 1 million generations, and posterior probability (PP) values were estimated with the remaining 75% of trees after a burn-in phase. The consensus trees were viewed in FigTree v. 1.4.4 (http://tree.bio.ed.ac.uk/software/figtree/ (accessed on 28 December 2023)).

## 3. Results

To determine the phylogenetic positions of the new species, single-gene datasets (BenA, CaM and RPB2) and concatenated ones were compiled and analyzed for *Penicillium* subgen. *Penicillium* sect. *Robsamsonia* ser. *Robsamsonia*, subgen. *Aspergilloides* sect. *Aspergilloides* ser. *Glabra*, and sect. *Citrina* ser. *Sumatraensia*. Detailed characteristics of the datasets are listed in Table 4.

**Figure 1:**
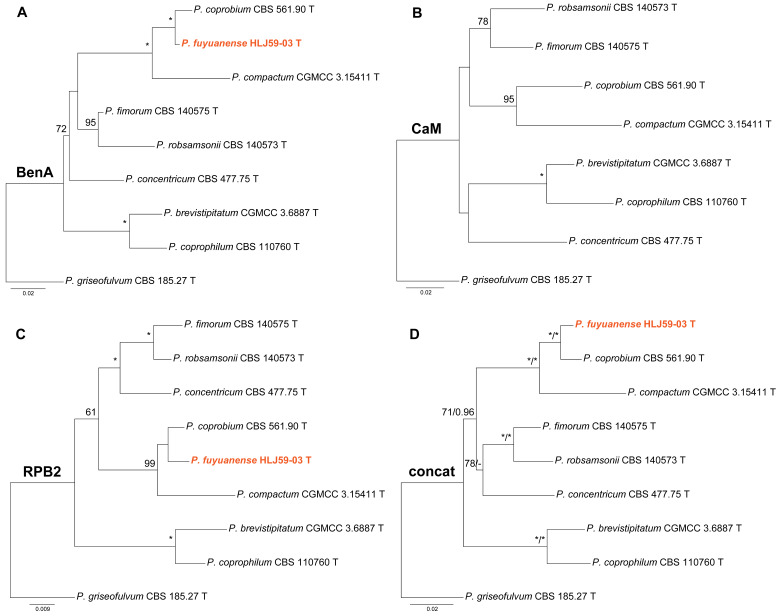
Maximum likelihood phylogeny of *Penicillium* subgen. *Penicillium* section *Robsamsonia* series *Robsamsonia* inferred from (**A**) BenA, (**B**) CaM, (**C**) RPB2 and (**D**) concatenated datasets. Bootstrap values ≥ 50% are indicated at nodes of (**A**–**C**); bootstrap values ≥ 50% (left) or posterior probability values ≥ 0.90 (right) are indicated at nodes of (**D**). Asterisk denotes 100% bootstrap or 1.00 posterior probability. The new species is in color.

Phylogenies of *Penicillium* subgen. *Penicillium* sect. *Robsamsonia* ser. *Robsamsonia* are given in Figure 1. In the BenA and concatenated phylogenies (Figure 1A,D), the strain HLJ59-03 was closely related to *P. coprobium* with strong support (MLBP = 100% or BIPP = 1.00). Although HLJ59-03 was also a sister taxon of *P. coprobium* in the ML tree based on RPB2 sequences (Figure 1C), the statistical support between them was very weak. HLJ59-03 was absent in the CaM phylogeny (Figure 1B) because of the failure of PCR amplification.

**Figure 2:**
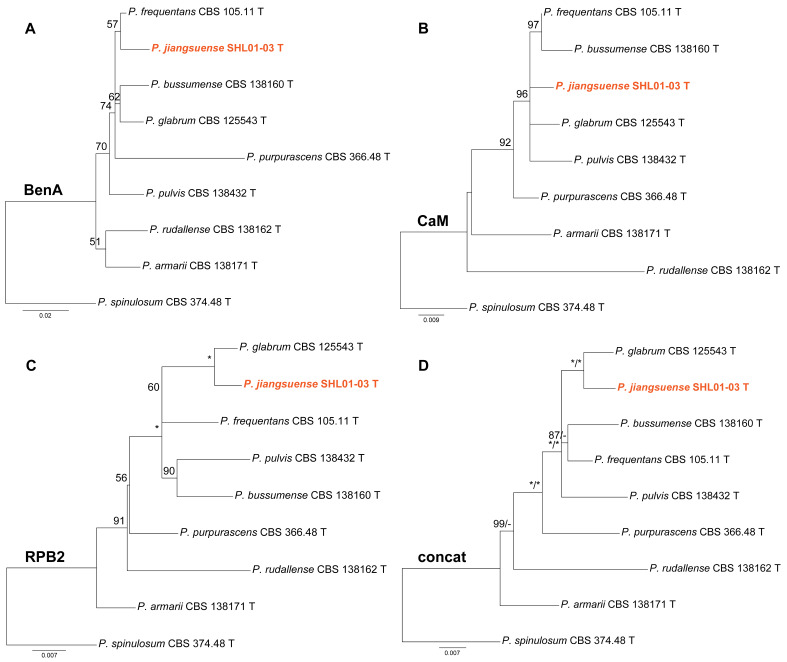
Maximum likelihood phylogeny of *Penicillium* subgen. *Aspergilloides* section *Aspergilloides* series *Glabra* inferred from (**A**) BenA, (**B**) CaM, (**C**) RPB2 and (**D**) concatenated datasets. Bootstrap values ≥ 50% are indicated at nodes of (**A**–**C**); bootstrap values ≥ 50% (left) or posterior probability values ≥ 0.90 (right) are indicated at nodes of (**D**). Asterisk denotes 100% bootstrap or 1.00 posterior probability. The new species is in color.

Phylogenies of *Penicillium* subgen. *Aspergilloides* sect. *Aspergilloides* ser. *Glabra* are shown in Figure 2. In the RPB2 and concatenated phylogenies (Figure 2C,D), strain SHL01-03 clustered with *P. glabrum* receiving strong support (MLBP = 100% or BIPP = 1.00). In contrast, SHL01-03 was a sister taxon of *P. frequentans* with weak support in the ML tree of BenA sequences (Figure 2A) and as an independent lineage in the CaM phylogeny (Figure 2B).

**Figure 3:**
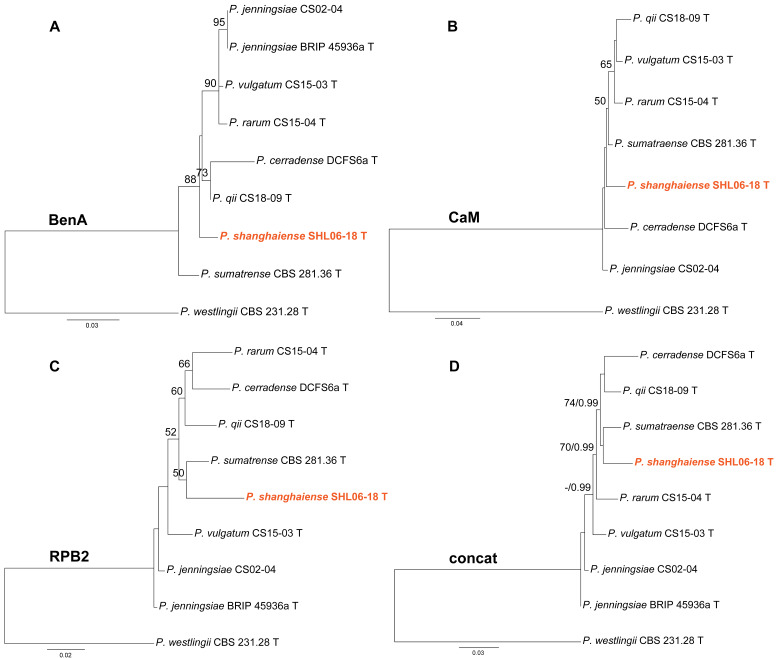
Maximum likelihood phylogeny of *Penicillium* subgen. *Aspergilloides* section *Citrina* series *Sumatraensia* inferred from (**A**) BenA, (**B**) CaM, (**C**) RPB2 and (**D**) concatenated datasets. Bootstrap values ≥ 50% are indicated at nodes of (**A**–**C**); bootstrap values ≥ 50% (left) or posterior probability values ≥ 0.90 (right) are indicated at nodes of (**D**). The new species is in color.

Phylogenies of *Penicillium* subgen. *Aspergilloides* sect. *Citrina* ser. *Sumatraensia* were depicted in Figure 3. Strain SHL06-18 was shown as an independent lineage in the BenA and CaM phylogenies (Figure 3A,B), while it was clustered with *P. sumatraense* in the RPB2 analysis and the concatenated ML tree (Figure 3C,D).

## 4. Taxonomy

**Figure 4:**
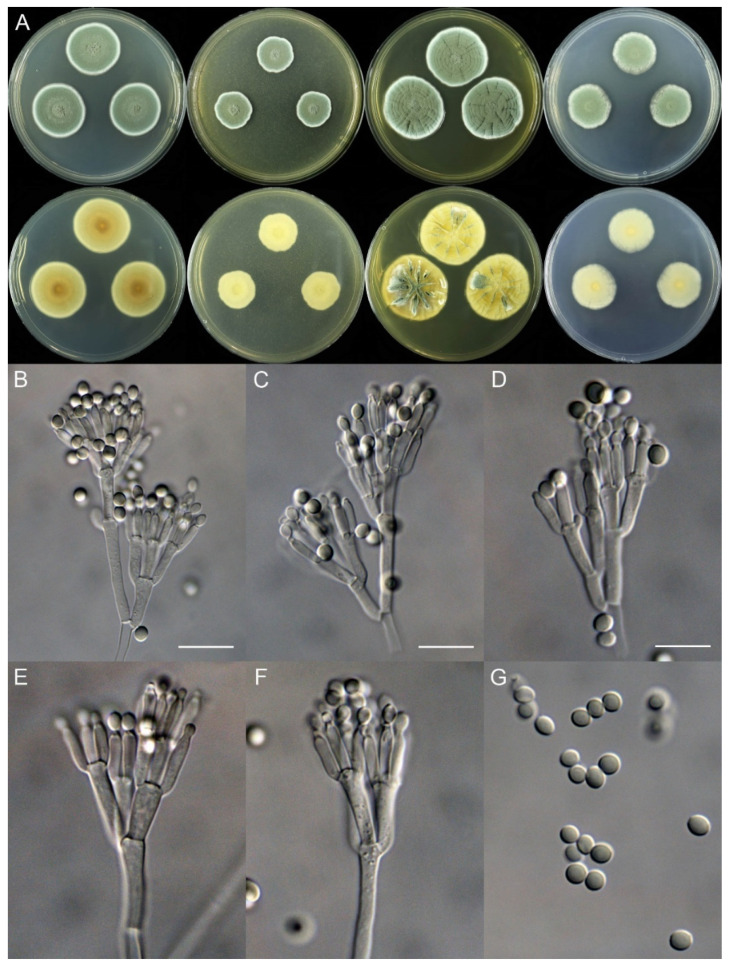
*Penicillium fuyuanense* (HLJ59-03). (**A**) Colonies: top row left to right, obverse CYA, MEA, YES, and PDA; bottom row left to right, reverse CYA, MEA, YES, and PDA; (**B**–**F**) Conidiophores; (**G**) Conidia. Bars: (**B**) = 15 µm; (**C**) = 12.5 µm; (**D**) = 10 µm, also for (**E**–**G**).

***Penicillium fuyuanense*** X.C. Wang & W.Y. Zhuang, sp. nov. Figure 4.

Fungal Names: FN571813.

Etymology: The specific epithet refers to the type locality.

In *Penicillium* subgenus *Penicillium* section *Robsamsonia* series *Robsamsonia*.

Typification: China. Heilongjiang Province, Jiamusi City, Fuyuan City, Fuyuan Town, Dongjige, 48°21′27″ N 134°16′58″ E, in soil under *Rhododendron dauricum* L., 14 May 2023, Xin-Cun Wang and He Song, culture, He Song, HLJ59-03 (holotype HMAS 247927, ex-type strain CGMCC 3.27293).

DNA barcodes: ITS PP357618, BenA PP373069, RPB2 PP373080.

Colony diam., 7 days, 25 °C (unless stated otherwise): CYA 27–28 mm; CYA 37 °C no growth; CYA 5 °C germinated, 3–4 mm; MEA 19–20 mm; YES 32–34 mm; PDA 22–23 mm.

Colony characteristics: On CYA 25 °C, 7 days: Colonies nearly circular, plain, protuberant and funiculose at centers, slightly concentrically sulcate; margins narrow, entire; mycelia white; texture velutinous; sporulation dense; conidia *en masse* dull green; soluble pigments absent; exudates absent; reverse yellow brown, white at margins.

On MEA 25 °C, 7 days: Colonies irregular, plain, protuberant and funiculose at centers; margins narrow, entire; mycelia white; texture velutinous; sporulation dense; conidia *en masse* dull green; soluble pigments absent; exudates absent; reverse white to buff, darker at centers.

On YES 25 °C, 7 days: Colonies nearly circular, plain or protuberant, concentrically and radially sulcate, funiculose at centers; margins narrow, entire; mycelia white; texture velutinous; sporulation dense; conidia *en masse* dull green; soluble pigments absent; exudates absent; reverse yellow brown.

On PDA 25 °C, 7 days: Colonies nearly circular to irregular, slightly protuberant at centers; margins narrow, entire or irregular; mycelia white; texture velutinous; sporulation dense; conidia *en masse* dull green; soluble pigments absent; exudates absent; reverse white, yellow at centers.

Micromorphology: Conidiophores biverticillate, terverticillate, quaterverticillate or more branched; stipes smooth-walled, 20–375 × 2.0–3.5 μm; branches 2, 16.5–28.5 × 2.5–5 μm; rami 2–3, 10–14.5 × 2.5–5.5 μm; metulae 2–5, 8–14.5 × 2.5–5 μm; phialides acerose, tapering into very thin neck, 4–6 per metula, 7.5–11 × 2.0–3.0 μm; conidia subglobose to ellipsoidal, smooth-walled, 3.5–4.5 × 3.0–4.0 μm.

Notes: This species is closely related to *P. coprobium* and *P. compactum* (Figure 1) phylogenetically. It differs from *P. coprobium* in 10 bp for BenA and 13 bp for RPB2, and from *P. compactum* in 25 bp for BenA and 29 bp for RPB2. Morphologically, it differs from *P. coprobium* in lacking of white sclerotia on MEA and producing quaterverticillate or even more branched conidiophores, shorter rami (10–14.5 vs. 12–20 μm), and larger conidia (3.5–4.5 × 3.0–4.0 vs. 3.2–4.0 × 2.5–3.0 μm) [26]. It differs from *P. compactum* in yellow brown other than dark brown or blackish brown colony on reverse view of CYA and YES at 25 °C, quaterverticillate or rich-branched conidiophores, longer stipes (20–375 vs. 40–100 μm) and shorter phialides (7.5–11 vs. 9–13 μm) [27]. Their morphological distinctions are summarized in Table 5.

**Figure 5:**
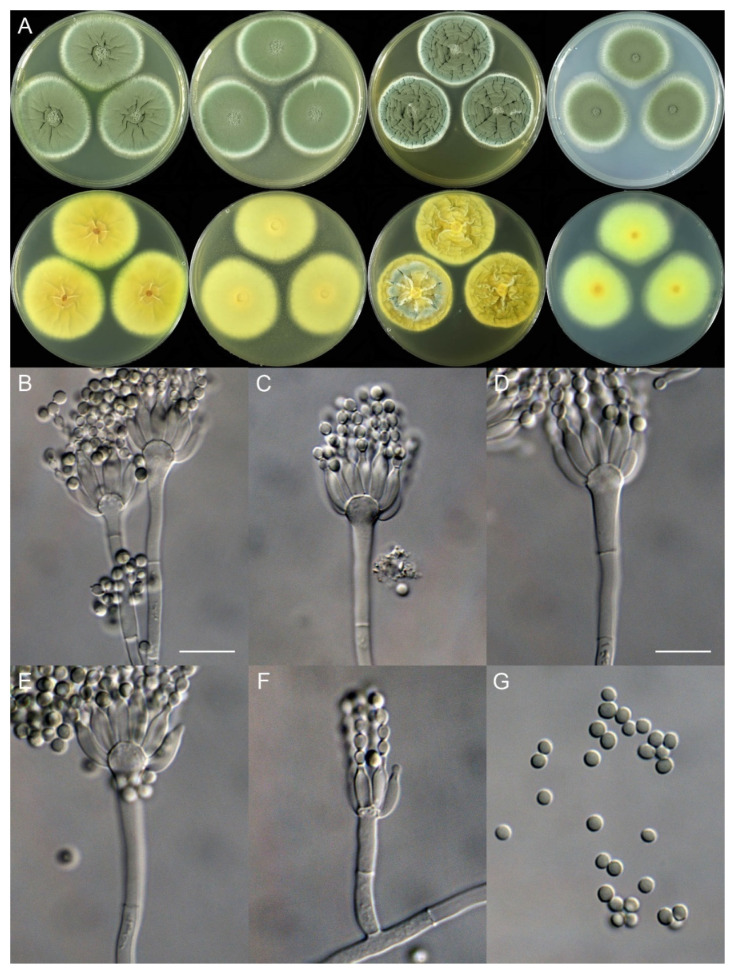
*Penicillium jiangsuense* (SHL01-03). (**A**) Colonies: top row left to right, obverse CYA, MEA, YES, and PDA; bottom row left to right, reverse CYA, MEA, YES, and PDA; (**B**–**F**) Conidiophores; (**G**) Conidia. Bars: (**B**) = 12.5 µm, also for (**C**); (**D**) = 10 µm, also for (**E**–**G**).

***Penicillium jiangsuense*** X.C. Wang & W.Y. Zhuang, sp. nov. Figure 5.

Fungal Names: FN571814.

Etymology: The specific epithet refers to the type locality.

In *Penicillium* subgenus *Aspergilloides* section *Aspergilloides* series *Glabra*.

Typification: China. Jiangsu Province, Lianyungang City, Haizhou District, Huaguoshan Mountain, 34°38′22″ N 119°16′55″ E, in soil, 20 April 2021, Xin-Cun Wang, culture, Yi-Jing Ding, SHL01-03 (holotype HMAS 247928, ex-type strain CGMCC 3.27294).

DNA barcodes: ITS PP357619, BenA PP373070, CaM PP373075, RPB2 PP373081.

Colony diam., 7 days, 25 °C (unless stated otherwise): CYA 44–45 mm; CYA 37 °C 8–9 mm; CYA 5 °C germinated, 2–3 mm; MEA 42–43 mm; YES 40–41 mm; PDA 40–41 mm.

Colony characteristics: On CYA 25 °C, 7 days: Colonies nearly circular, protuberant at centers, radially sulcate; margins moderately wide, entire; mycelia white; texture velutinous; sporulation dense; conidia *en masse* dull green; soluble pigments absent; exudates absent; reverse yellow brown, red brown at centers.

On CYA 37 °C, 7 days: Colonies irregular, protuberant, cerebroid, strongly sulcate; margins narrow, irregular; mycelia white; texture tight; sporulation absent; conidia *en masse* not seen; soluble pigments absent; exudates absent; reverse carneous or flesh-colored, lighter at centers.

On MEA 25 °C, 7 days: Colonies nearly circular, plain, funiculose at centers; margins moderately wide, entire; mycelia white; texture velutinous; sporulation dense; conidia *en masse* dull green; soluble pigments absent; exudates absent; reverse light yellow brown.

On YES 25 °C, 7 days: Colonies nearly circular, strongly sulcate, concave at centers; margins narrow to moderately wide, entire; mycelia white; texture velutinous; sporulation dense; conidia *en masse* dull green; soluble pigments absent; exudates absent; reverse yellow brown.

On PDA 25 °C, 7 days: Colonies nearly circular, plain, slightly funiculose at centers; margins wide, fimbriate; mycelia white; texture velutinous; sporulation dense; conidia *en masse* dull green; soluble pigments absent; exudates absent; reverse white, orange brown at centers.

Micromorphology: Conidiophores monoverticillate; stipes smooth-walled, rarely rough-walled, 35–85 × 2.5–4.0 μm; phialides ampulliform, tapering into a very thin neck, 8–12 per metula, 8.5–11 (–13.5) × 2.5–4.5 μm; conidia subglobose to ellipsoidal, smooth-walled, 3.0–3.5 × 2.5–3.0 μm.

Notes: This species is closely related to *P. frequentans* (Figure 2A) and *P. glabrum* phylogenetically (Figure 2C,D). It differs from *P. frequentans* in 7 bp for BenA, 5 bp for CaM and 22 bp for RPB2; and from *P. glabrum* in 10 bp for BenA, 8 bp for CaM and 10 bp for RPB2. Morphologically, it differs from the above two species in being able to grow on CYA at 37 °C and having smooth-walled and slightly larger conidia (3.0–3.5 vs. 2.5–3.0 μm) [28,29]. Their morphological distinctions are listed in Table 6.

**Figure 6:**
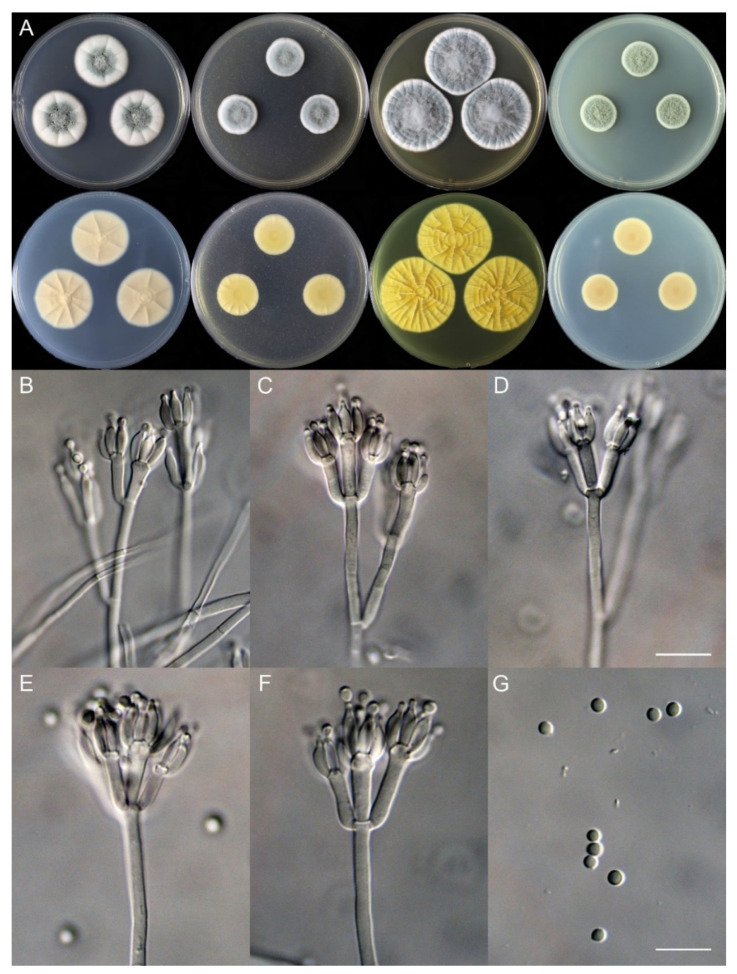
*Penicillium shanghaiense* (SHL06-18). (**A**) Colonies: top row left to right, obverse CYA, MEA, YES, and PDA; bottom row left to right, reverse CYA, MEA, YES, and PDA; (**B**–**F**) Conidiophores; (**G**) Conidia. Bars: (**D**) = 12.5 µm, also for (**B**,**C**); (**G**) = 10 µm, also for (**E**,**F**).

***Penicillium shanghaiense*** X.C. Wang & W.Y. Zhuang, sp. nov. Figure 6.

Fungal Names: FN571815.

Etymology: The specific epithet refers to the type locality.

In *Penicillium* subgenus *Aspergilloides* section *Citrina* series *Sumatraensia*.

Typification: China. Shanghai City, Chongming District, Dongtan National Nature Reserve, 31°31′6″ N 121°56′58″ E, in soil under *Camphora officinarum* Nees ex Wall., 23 April 2021, Xin-Cun Wang, culture, Yi-Jing Ding, SHL06-18 (holotype HMAS 247929, ex-type strain CGMCC 3.27295).

DNA barcodes: ITS PP357620, BenA PP373071, CaM PP373076, RPB2 PP373082.

Colony diam., 7 days, 25 °C (unless stated otherwise): CYA 29–31 mm; CYA 37 °C no growth; CYA 5 °C no growth; MEA 20–21 mm; YES 37–39 mm; PDA 18–19 mm.

Colony characteristics: On CYA 25 °C, 7 days: Colonies nearly circular, slightly protuberant at centers, radially sulcate; margins wide, entire; mycelia white; texture velutinous; sporulation moderately dense; conidia *en masse* bluish green to dull green; soluble pigments absent; exudates abundant, clear; reverse carneous or flesh-colored.

On MEA 25 °C, 7 days: Colonies nearly circular, protuberant; margins narrow to moderately wide, entire; mycelia white; texture floccose; sporulation dense; conidia *en masse* greyish green; soluble pigments absent; exudates absent; reverse yellow brown, buff at margins.

On YES 25 °C, 7 days: Colonies nearly circular, strongly sulcate; margins narrow to moderately wide, undulated; mycelia white; texture floccose, velutinous at margin areas; sporulation dense; conidia *en masse* greyish green; soluble pigments absent; exudates absent; reverse buff to yellow brown.

On PDA 25 °C, 7 days: Colonies nearly circular; margins narrow, entire; mycelia white; texture floccose; sporulation dense; conidia *en masse* greyish green; soluble pigments absent; exudates absent; reverse light brown.

Micromorphology: Conidiophores biverticillate or terverticillate; stipes smooth-walled, 85–415 × 2.0–3.0 μm; rami 2, 21.5–32.5 × 2.0–3.0 μm; metulae 2–5, 9.5–17.5 × 2.0–5.0 μm; phialides ampulliform, tapering into a very thin neck, 3–8 per metula, 6.0–9.0 × 2.0–3.0 μm; conidia subglobose, smooth-walled, 2.0–3.0 μm.

Notes: This species is a member of the series *Sumatraensia* and a sister of the other six species in the group (Figure 3). Phylogenetically, it differs from *P. cerradense* in 97 bp for the three gene fragments (the details can be found in Table 7), from *P. jenningsiae* in 71 bp, from *P. qii* in 66 bp, from *P. rarum* in 76 bp, from *P. sumatraense* in 72 bp, and from *P. vulgatum* in 69 bp. Morphologically, the new species differs from *P. cerradense* in terms of its faster growth rate on MEA (20–21 vs. 15 mm), slower growth rate on PDA (18–19 vs. 30 mm), terverticillate instead of predominantly monoverticillate conidiophores, and absence of sclerotia [30]; from *P. jenningsiae* in slower growth rates on the four media, terverticillate conidiophores, longer stipes (85–415 vs. 100–250 μm), and shorter phialides (6–9 vs. 8–12 μm) [12,31]; from *P. qii* in terverticillate conidiophores [12]; from *P. rarum* in without monoverticillate conidiophores and longer rami (21.5–32.5 vs. 14–20 μm) [12]; from *P. sumatraense* in its slower growth rate on MEA (20–21 vs. 30–45 mm) [29]; and from *P. vulgatum* in its slower growth rates on the four media and terverticillate conidiophores [12]. Table 7 provides the detailed distinctions among taxa in the series.

## 5. Discussion

The three new species were isolated from soil samples collected from East China (Jiangsu Province and Shanghai City) and Northeast China (Heilongjiang Province). They are morphologically and phylogenetically distinguishable from any existing species of the genus and belong to the *Penicillium* subgenus *Penicillium* section *Robsamsonia* series *Robsamsonia*, subgenus *Aspergilloides* section *Aspergilloides* series *Glabra* and section *Citrina* series *Sumatraensia*.

The three new species are from different climate zones. *Penicillium fuyuanense*, along with its closest sisters *P. coprobium* and *P. compactum*, are all from boreal (cold–temperate) zone and high-latitude areas. *Penicillium jiangsuense*, *P. shanghaiense* and their relatives are from a temperate zone. Most species of *Penicillium* are usually found in the same climate zone. *Penicillium choerospondiatis* X.C. Wang & W.Y. Zhuang occurred in subtropical China [15], and was later reported to be found in Kolkata, India [32]. For the time being, it seems that samples of individual new species are expected to be seen in similar climates.

Northeast China and East China encompass 10 provinces and Shanghai Municipal City, accounting for 17% area of this country, in which nine *Penicillium* species were originally described, e.g., *P. compactum* from Heilongjiang, *P. jianxiense* H.Z. Kong & Z.Q. Liang from Jiangxi, and *P. formosanum* H.M. Hsieh, H.J. Su & Tzean, and *P. ulaiense* H.M. Hsieh, H.J. Su & Tzean from Taiwan. Compared with the fact that 37 new species of the genus have recently been recorded in Chongqing Municipal City [12], covering only 0.86% of China, more intensive investigations are desperately needed to extend our knowledge on the biodiversity of *Penicillium* in our country.

## Figures and Tables

**Table 1:** Species and sequences of *Penicillium* subgen. *Penicillium* sect. *Robsamsonia* ser. *Robsamsonia* used in phylogenetic analyses.

Series	Species	Strain	Locality	Substrate	ITS	BenA	CaM	RPB2
*Robsamsonia*	*P. brevistipitatum* L. Wang & W.Y. Zhuang 2005	CGMCC 3.6887 T	China: Jilin	soil	DQ221696	DQ221695	KU896824	JN406528
*Robsamsonia*	*P. compactum* L. Wang & Houbraken 2016	CGMCC 3.15411 T	China: Heilongjiang	soil	KM973207	KM973203	KM973200	KT698909
*Robsamsonia*	*P. concentricum* Samson et al., 1976	CBS 477.75 T	Germany	colon of Cervidae	KC411763	AY674413	DQ911131	KT900575
*Robsamsonia*	*P. coprobium* Frisvad 1990	CBS 561.90 T	Norway	pig feed	DQ339559	AY674425	KU896830	KT900576
*Robsamsonia*	*P. coprophilum* (Berk. & M.A. Curtis) Seifert & Samson 1986	CBS 110760 T	Cuba	dung of Aves	AF033469	AY674421	KU896831	JN406645
*Robsamsonia*	*P. fimorum* Houbraken & Frisvad 2016	CBS 140575 T	Denmark	dung of mouse	KU904343	KT698889	KT698898	KT698908
*Robsamsonia*	*P. fuyuanense* X.C. Wang & W.Y. Zhuang, sp. nov.	HLJ59-03 = CGMCC 3.27293 T	China: Heilongjiang	soil under *Rhododendron dauricum*	**PP357618**	**PP373069**	n.a.	**PP373080**
*Robsamsonia*	*P. robsamsonii* Houbraken & Frisvad 2016	CBS 140573 T	Denmark	dung of mouse	KU904339	KT698885	KT698894	KT698904
*Urticicolae*	*P. griseofulvum* Dierckx 1901	CBS 185.27 T	unknown	unknown	AF033468	JF909942	KT900574	JN121449

GenBank accession numbers in bold indicate the newly generated sequences. The phrase ‘n.a.’ is the abbreviation of ‘not available’.

**Table 2:** Species and sequences of *Penicillium* subgen. *Aspergilloides* sect. *Aspergilloides* ser. *Glabra* used in phylogenetic analyses.

Series	Species	Strain	Locality	Substrate	ITS	BenA	CaM	RPB2
*Glabra*	*P. armarii* Houbraken et al., 2014	CBS 138171 T	Australia	house dust	KM189758	KM089007	KM089394	KM089781
*Glabra*	*P. bussumense* Houbraken 2014	CBS 138160 T	The Netherlands	soil	KM189458	KM088685	KM089070	KM089457
*Glabra*	*P. frequentans* Westling 1911	CBS 105.11 T	unknown	unknown	KM189525	KM088762	KM089147	KM089534
*Glabra*	*P. glabrum* (Wehmer) Westling 1911	CBS 125543 T	unknown	unknown	GU981567	GU981619	KM089152	JF417447
*Glabra*	*P. jiangsuense* X.C. Wang & W.Y. Zhuang, sp. nov.	SHL01-03 = CGMCC 3.27294 T	China: Jiangsu	soil	**PP357619**	**PP373070**	**PP373075**	**PP373081**
*Glabra*	*P. pulvis* Houbraken et al., 2014	CBS 138432 T	South Africa	house dust	KM189632	KM088876	KM089263	KM089650
*Glabra*	*P. purpurescens* (Sopp) Biourge 1923	CBS 366.48 T	Canada	soil	KM189561	KM088801	KM089186	KM089573
*Glabra*	*P. rudallense* Houbraken et al., 2014	CBS 138162 T	Australia	soil	KM189504	KM088741	KM089126	KM089513
*Spinulosa*	*P. spinulosum* Thom 1910	CBS 374.48 T	Germany	culture contaminant	AF033410	KJ834493	GQ367524	JN406558

GenBank accession numbers in bold indicate the newly generated sequences.

**Table 3:** Species and sequences of *Penicillium* subgen. *Aspergilloides* sect. *Citrina* ser. *Sumatraensia* used in phylogenetic analyses.

Series	Species	Strain	Locality	Substrate	ITS	BenA	CaM	RPB2
*Sumatraensia*	*P. cerradense* Cruvinel et al., 2021	DCFS6a T	Brazil	soil	MT006126	MT416533	MT416534	MT416532
*Sumatraensia*	*P. jenningsiae* Y.P. Tan et al., 2022	BRIP 45936a T	Australia	compost	n.a.	OL741657	n.a.	OL741660
		CS02-04	China: Chongqing	soil	OQ870876	OR051078	OR051255	OR051429
*Sumatraensia*	*P. qii* X.C. Wang & W.Y. Zhuang 2023	CS18-09 = CGMCC 3.25165 T	China: Chongqing	soil	OQ870878	OR051080	OR051257	OR051430
*Sumatraensia*	*P. rarum* X.C. Wang & W.Y. Zhuang 2023	CS15-04 = CGMCC 3.25166 T	China: Chongqing	soil	OQ870881	OR051083	OR051260	OR051432
*Sumatraensia*	*P. shanghaiense* X.C. Wang & W.Y. Zhuang, sp. nov.	SHL06-18 = CGMCC 3.27295 T	China: Shanghai	soil under *Camphora officinarum*	**PP357620**	**PP373071**	**PP373076**	**PP373082**
*Sumatraensia*	*P. sumatraense* Svilv. 1936	CBS 281.36 T	Indonesia	heath soil	GU944578	JN606639	MN969301	EF198541
*Sumatraensia*	*P. vulgatum* X.C. Wang & W.Y. Zhuang 2023	CS15-03 = CGMCC 3.25180 T	China: Chongqing	soil	OQ870884	OR051086	OR051263	OR051434
*Westlingiorum*	*P. westlingii* K.W. Zaleski 1927	CBS 231.28 T	Poland	soil under conifer	GU944601	JN606718	MN969312	JN606625

GenBank accession numbers in bold indicate the newly generated sequences. The phrase ‘n.a.’ is the abbreviation of ‘not available’.

**Table 4:** Detailed characteristics of the datasets.

Dataset	Gene Fragment	No. of Seq.	Length of Alignment (bp)	No. of Variable Sites	No. of Parsimony-Informative Sites	Model for BI
*Robsamsonia*	BenA	9	436	95	43	
	CaM	8	510	114	51	
	RPB2	9	915	150	75	
	BenA+CaM+RPB2	9	1861	359	169	SYM+G
*Glabra*	BenA	9	418	67	16	
	CaM	9	486	82	18	
	RPB2	9	885	96	32	
	BenA+CaM+RPB2	9	1789	245	66	SYM+G
*Sumatraensia*	BenA	9	445	73	20	
	CaM	8	569	132	12	
	RPB2	9	915	142	39	
	BenA+CaM+RPB2	9	1929	347	71	TrNef+I+G

Abbreviations of models: SYM+G (symmetrical model with Gamma distribution); TrNef+I+G (equal-frequency Tamura–Nei model with invariant sites and Gamma distribution).

**Table 5:** Morphological comparisons of new species and their closely related species in series *Robsamsonia*.

Species	CYA 25 °C (mm)	CYA 37 °C (mm)	MEA (mm)	YES (mm)	Conidiophore	Conidia Shape	Conidia Wall	Conidia Size (µm)	Reference
*P. fuyuanense*	27–28	no growth	19–20	32–34	biverticillate to quaterverticillate	subglobose to ellipsoidal	smooth	3.5–4.5 × 3.0–4.0	This study
*P. compactum*	17–23	no growth	22–28	29–35	terverticillate	ellipsoidal	smooth	4.0–4.5 × 3.5–4.0	[27]
*P. coprobium*	20–26	n.a.	n.a.	29–39	terverticillate	broadly ellipsoidal	smooth	3.2–4.0 × 2.5–3.0	[26,27]

**Table 6:** Morphological comparisons of new species and their closely related species in the series *Glabra*.

Species	CYA 25 °C (mm)	CYA 37 °C (mm)	MEA (mm)	YES (mm)	Conidiophore	Conidia Shape	Conidia Wall	Conidia Size (µm)	Reference
*P. jiangsuense*	44–45	8–9	42–43	40–41	monoverticillate	subglobose to ellipsoidal	smooth	3.0–3.5 × 2.5–3.0	This study
*P. frequentans*	38–50	no growth	38–51	40–53	monoverticillate	globose to subglobose	finely rough to rough	2.5–3.0	[28,29]
*P. glabrum*	35–48	no growth	38–50	40–59	monoverticillate	globose to subglobose	finely rough	2.5–3.0	[28,29]

**Table 7:** Morphological and molecular comparisons of new species and their closely related species in the series *Sumatraensia*.

Species	CYA 25 °C (mm)	CYA 37 °C (mm)	MEA (mm)	YES (mm)	Conidiophore	Conidia Shape	Conidia Wall	Conidia Size (µm)	Reference	BenA Difference (bp)	CaM Difference (bp)	RPB2 Difference (bp)
*P. shanghaiense*	29–31	no growth	20–21	37–39	biverticillate or terverticillate	subglobose	smooth	2.0–3.0	This study	_	_	_
*P. cerradense*	n.a.	n.a.	15	n.a.	monoverticillate or biverticillate	subglobose or ellipsoidal	smooth	1.5–3.0 × 2.0–3.0	[30]	32	25	40
*P. jenningsiae*	37–38	no growth	29–30	42–43	divaricate or biverticillate	globose to subglobose	smooth	2.0–3.0	[12,31]	15	17	39
*P. qii*	31–32	no growth	19–20	37–38	biverticillate	subglobose to broad ellipsoidal	smooth	2.5–3.0	[12]	7	20	39
*P. rarum*	28–36	no growth	17–23	32–42	terverticillate, biverticillate or monoverticillate	subglobose	smooth	2.5–3.0	[12]	11	19	46
*P. sumatraense*	25–35	no growth	30–45	n.a.	terverticillate, biverticillate or monoverticillate	spheroidal to subspheroidal	smooth	2.5–3.0	[29]	17	18	37
*P. vulgatum*	34–35	no growth	23–24	42–43	biverticillate	subglobose to ellipsoidal	smooth	2.5–3.0 × 2.0–2.8	[12]	12	18	39

## Data Availability

Data are contained within the article.
